# Population-level Variability in Genome-wide Repressive Histone Marks in a Fungal Wheat Pathogen

**DOI:** 10.1093/gbe/evag118

**Published:** 2026-05-08

**Authors:** Leen Nanchira Abraham, Ana Margarida Sampaio, Suhani Bhattacharyya, Sabina Moser Tralamazza, Daniel Croll

**Affiliations:** Laboratory of Evolutionary Genetics, Institute of Biology, University of Neuchâtel, Neuchâtel 2000, Switzerland; Laboratory of Evolutionary Genetics, Institute of Biology, University of Neuchâtel, Neuchâtel 2000, Switzerland; Laboratory of Evolutionary Genetics, Institute of Biology, University of Neuchâtel, Neuchâtel 2000, Switzerland; Laboratory of Evolutionary Genetics, Institute of Biology, University of Neuchâtel, Neuchâtel 2000, Switzerland; Laboratory of Evolutionary Genetics, Institute of Biology, University of Neuchâtel, Neuchâtel 2000, Switzerland

**Keywords:** fungi, comparative genomics, epigenomics, histone methylation

## Abstract

Epigenetic modifications influence the expression of phenotypic traits by modulating gene expression and responses to environmental cues. In plant pathogens, the expression of virulence-associated genes is known to be regulated by epigenetic modifications and is considered a key adaptation for pathogens. Gene expression variation within pathogen species is regulated by extensive cis*-*regulatory polymorphism and insertion activities of transposable elements. However, whether pathogens vary in epigenetic profiles among members of the same species remains largely unexplored. Here, we focus on the major fungal wheat pathogen *Zymoseptoria tritici* and establish histone methylation profiles for 45 isolates of an extensively characterized wheat field population. We analyzed the facultative heterochromatin mark H3K27me3, thought to regulate effector and gene cluster loci in the genome. H3K27m3 coverage was increased in transposable element-rich regions, with newly inserted retrotransposons contributing to epigenetic variation among pathogen genotypes. Nearly 20% of all genes showed within-population variation in H3K27me3 marks, which likely contributes to the substantial within-population variation in gene expression. Effector candidate genes and members of gene clusters showed higher than average H3K27me3 variation. Our study provides among the first insights into intra-species epigenetic variation of a fungal pathogen and opens avenues to recapitulate epigenetic mechanisms of pathogen adaptation.

Significance statement Epigenetic changes can regulate gene activity without altering DNA and play a key role in how organisms respond to their environment. In plant pathogens, these changes can control crucial genes for host infection. Whether such epigenetic marks are variable within species remains poorly understood, though. To address this, we investigated field-collected isolates of the wheat pathogen *Zymoseptoria tritici* to explore variation in histone methylation across the genome. We found that transposable elements often shape these marks and that nearly 20% of genes vary in their histone methylation profiles. This provides strong support that epigenetic variation constitutes an important factor of diversity within pathogen species and that this variation may facilitate adaptation.

## Introduction

Phenotypic variation among individuals may arise from mutations or nongenetic changes (Schmitz et al. 2013). Major epigenetic processes, including DNA methylation, histone modification, and various RNA-mediated processes, regulate spatial and temporal gene expression patterns (Gibney and Nolan 2010). Among these, covalent modifications of histones modify the local chromatin structure and affect DNA accessibility to transcription factors regulating gene expression (Gibney and Nolan 2010). Chromatin is organized by the nucleosome, an octamer with 147 base pairs of DNA wrapped around histone proteins. Histone proteins (H2A, H2B, H3, and H4) are made up of a globular domain and an unstructured tail domain that can be modified by acetylation, methylation, phosphorylation, and ubiquitylation (Bannister and Kouzarides 2011). Among these, methylation of histone tails is well studied and has diverse important biological functions (Martin and Zhang 2005). Methylation of histone H3 at lysine 4 residue (H3K4me) is a broadly found mark for transcriptionally active and gene-dense regions in eukaryotes. Although many aspects of H3K4me mechanisms and functions appear to be shared among kingdoms, there are significant differences between H3K4me and transcription in plants and mammals. For instance, plants appear to lack preferential co-localization of H3K4me3 and H3K27 as has been shown in mammals. Similarly, H3K4me2/3 and DNA methylation seem to be mutually exclusive in *Arabidopsis thaliana* (Zhang et al. 2009). Methylation of histone H3 at lysine 9 residue (H3K9me) is typically found in repeat-rich regions near transposable elements and satellite repeats, causing transcriptional silencing (Kabi and Filion 2021). In addition to heterochromatin formation, H3K9 methylation is a prerequisite for DNA methylation, another type of epigenetic modification that is involved in gene silencing (Tamaru and Selker 2001; Jackson et al. 2002). Comparative analyses of DNA methylation in eukaryotes showed that budding and fission yeasts are devoid of DNA methylation (O’Kane and Hyland 2019). *Saccharomyces cerevisiae* also lacks repressive histone H3K9 methylation, suggesting yeast lineages lost this epigenetic pathway (Hansen et al. 2011). The trimethylation of histone H3 at lysine 27 (H3K27) modification underpins facultative heterochromatin and is associated with regions of responsive gene expression regulation (Jamieson et al. 2013). Unlike H3K9 methylation, which prevents the binding of transcription factors and causes a persistent state of silencing, H3K27 allows genes to be activated through transcription factor binding in response to environmental cues and stress (Bannister and Kouzarides 2011; Tsompana and Buck 2014; Cai et al. 2021). Despite extensive knowledge of gene functions and domains preferentially associated with different chromatin states, how chromosomal regions gain specific marks remains poorly understood.

Histone methylation marks are not conserved within species and epigenetic variation can underpin gene expression variation (Johnson and Tricker 2010). In *A. thaliana*, H3K27 occupancy varies little, and the flanking transposable elements appear to account for most variation (Dong et al. 2012). In humans, histone tail modifications are highly variable but stably inherited across generations (Kasowski et al. 2013). Hundreds of sequence variants in the human genome are identified to be associated with both histone modification and gene expression variation consistent with widespread epigenetic effects on gene expression (McVicker et al. 2013). DNA-binding molecules that bind to specific DNA motifs can recruit or stabilize histone modifications (Whitaker et al. 2014). In humans and rats, histone-associated DNA motifs have shown significant overlap with the expression of quantitative trait loci SNPs, suggesting an important role in gene regulation (Ngo et al. 2019). Histone methylation levels vary in rat genomes in response to cis- and trans*-*acting regulatory factors, indicating that histone trimethylation marks are impacted by genetic variation (Rintisch et al. 2014). Insights from these studies strongly suggest that epigenetic variation in histone methylation marks is widespread within species and likely affects adaptive phenotypic trait variation. However, studies on the extent and consequences of histone methylation polymorphism in natural populations are lacking, with few exceptions (Johnston et al. 2022).

In fungi, epigenetic modifications can facilitate host evasion by enabling flexible gene regulation, such as the repression of pathogenicity-related genes during vegetative growth and their rapid activation during infection (Soyer et al. 2014; He et al. 2020). Plant pathogens need to rapidly respond to environmental cues when in contact with a plant host (Braunsdorf et al. 2016). Plants produce a variety of molecules to inhibit fungal development (Ferreira et al. 2007). In turn, fungal pathogens secrete secondary metabolites and small proteins (ie effectors) to manipulate the host (Macheleidt et al. 2016). Up-regulation of pathogen genes upon infection is highly concerted and characterized by an initial wave of effector genes and specialized metabolite gene cluster expression (Jeon et al. 2015; Palma-Guerrero et al. 2016; Qiu et al. 2016; Ramírez-Tejero et al. 2020; Ibrahim et al. 2021; W. Zhang et al. 2021a; Kramer et al. 2022). Regulatory control of some effector genes is governed by epigenetic changes related to H3K9me3 marks, such as in the rapeseed pathogen *Leptosphaeria maculans (Soyer et al. 2014).* Effector genes residing in similarly repeat-rich and repressive regions in the wheat pathogen *Zymoseptoria tritici* are upregulated by a reduction in H3K9me3 and H3K27 (Meile et al. 2020). Infection-related metabolite gene clusters are largely regulated epigenetically with frequent associations with H3K27 in the fungal pathogens *Epichloë festucae, Fusarium graminearum, F. fujikuroi, and Colletotrichum higginsianum (O’Connell et al. 2012; Visentin et al. 2012; Connolly et al. 2013; Winter et al. 2018)*. These results suggest that histone methylation may dynamically modulate multiple pathogenicity traits, enabling reversible changes in effector expression and secondary metabolite production. This may have consequences for the durability and effectiveness of disease management strategies. Histone methylation also plays broader roles in the evolution of fungi, with H3K27 marks underpinning reduced transcriptional robustness (Tralamazza et al. 2022) and the loss of H3K27 contributing to elevated mutation rates (Habig et al. 2021).

The wheat pathogen *Z. tritici* causes severe yield losses under conducive conditions and has spread globally with the introduction of the host (Feurtey et al. 2023). Gene regulation is governed by numerous expression quantitative trait loci (eQTLs) located close to transcription start sites (Abraham and Croll 2023). The genome is organized into distinct compartments of gene-dense regions of open chromatin and repeat-rich regions with repressive H3K9me3 and H3K27 marks (Schotanus et al. 2015). TEs actively create new copies, reshaping the genomic landscape, impacting gene expression, phenotypic trait variation, and genome size (Fouché et al. 2020; Oggenfuss et al. 2021; Singh et al. 2021b; Oggenfuss and Croll 2023; Baril et al. 2025).

The species has lost a functional DNA methylation machinery in its recent evolutionary history, though (Moller et al. 2021). H3K9me3 and H3K27 histone methylation marks affect negatively and positively the base mutation rate, respectively (Habig et al. 2021). Strikingly, H3K9me3 supports and H3K27 reduces the stability of degenerated accessory chromosomes (Möller et al. 2019). The species carries vast polymorphism at the genetic and transcriptional levels (Palma-Guerrero et al. 2016; Hartmann et al. 2017; Palma-Guerrero et al. 2017; Singh et al. 2021a, 2021b; Abraham and Croll 2023; Feurtey et al. 2023). Gene regulation is governed by cis and trans*-*acting loci, with most genes showing evidence for at least one regulatory region (Abraham and Croll 2023). However, structural variation and movements of TEs likely also impact the epigenetic landscape (Fouché et al. 2020; Oggenfuss et al. 2021; Singh et al. 2021a; Oggenfuss and Croll 2023) with consequences for the expression of individual genes.

Here, we generated genome-wide H3K27me3 profiles using chromatin immunoprecipitation sequencing (ChIP-seq) for a highly polymorphic population of 45 *Z. tritici* isolates. The experiments were conducted in a minimal culture medium simulating the early phase of infection. We first searched for genomic factors underpinning H3K27me3 variation among genotypes, such as gene density and recent TE activity. Then, we assessed the impact of H3K27me3 variation on gene expression and analyzed variation in histone methylation marks across gene functions encoded by the pathogen genome. We focused in particular on epigenetic variation near effector genes and secondary metabolite gene clusters.

## Results

### Population Chromatin Immunoprecipitation Sequencing (ChIP-Seq)

**Fig. 1.**
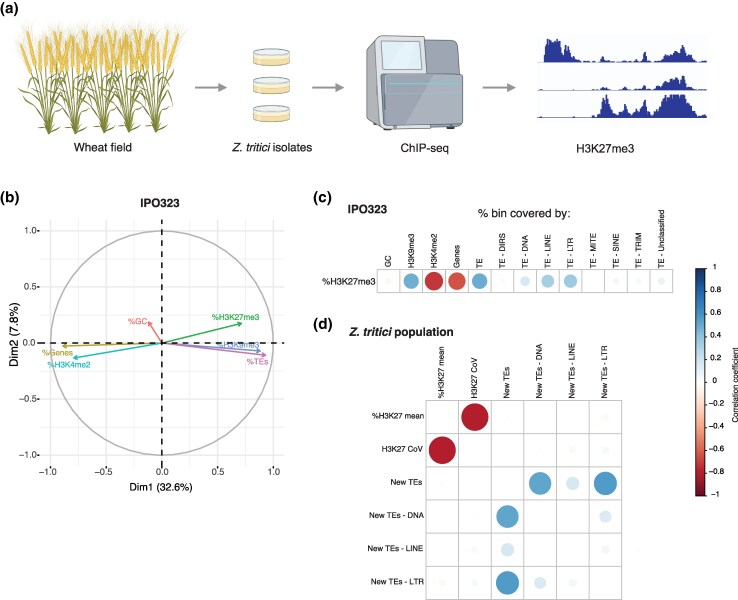
Experimental design and genomic features correlated with histone methylation mark variation. a) Schematic overview of the study design (created with BioRender.com). b) Ordination plot of GC content, H3K27m3, H3K9m3, H3K4m3 coverage, coding sequence density, and transposable element (TE) density in the reference genome. The metrics were assessed in genomic windows of 10 kb. c) Correlation plot between H3K27m3 coverage and GC content, H3K9m3, H3K4m2, coding sequences, and TEs (incl. superfamily categories) in the reference genome. d) Correlation plot between mean H3K27m3 coverage and the coefficient of variation among bins, as well as counts of recently inserted TEs (incl. superfamily categories) among isolates of the analyzed population.

We performed genome-wide ChIP-seq analyses of H3K27m3 marks in a highly diverse population of *Z. tritici* (*n* = 45) isolated from a naturally infected Swiss wheat field (Singh et al. 2021b) (Fig. 1a). We obtained between 339 and 985 Mb of sequencing data per isolate. The mapping rate of reads against the reference genome IPO323 ranges from 76.3% to 92.2%, comparable to mapping rates for whole genome sequencing data of the same population (Singh et al. 2021a) (Table S1, Fig. S1a). Between 54.2% to 76.7% of mapped reads were uniquely aligned to the reference genome (Fig. S1a). After duplicate filtering, we retained between 7 and 20 million mapped reads per isolate. We calculated the normalized strand cross-correlation coefficient (NSC) as an indicator of the H3K27 signal-to-background noise ratio. The NSC was consistently above 1.05, indicating adequate enrichment in H3K27m3 signals (Fig. S1b). We assessed read distribution biases across the genome and found background uniformity (Bu) values to be mainly above 0.8, indicating low false positive peak calling risks (Fig. S1c). The peak calling on the aligned H3K27m3 ChIP-seq reads produced between 1,571 and 9,428 peaks (average 2,953) among isolates (Fig. S1d) stemming from an average of 56% of all aligned reads (FRiP score; Fig. S1e). Input controls can vary substantially between biological replicates (Soyer et al. 2015), reducing their utility for capturing true population-level H3K27m3 variation. In the absence of input controls, H3K27m3 peak calling robustness was assessed by comparing predictions by 2 independent peak callers using distinct background modeling strategies. Overlap across isolates ranged from 63.5% to 92.8%, with a mean of 86.6% (Table S1). Hence, the reported peaks are broadly supported by independent peaking calling algorithms.

### Genomic Factors Associated With Histone Methylation Variation

To assess genomic factors associated with histone methylation variation among isolates, we first explored ChIP-seq peak distribution in the reference genome using 10 kb windows. For the first analyses, we focused on previously generated ChIP-seq data for the reference genome isolate IPO323 and the marks H3K27m3, H3K9me3, and H3K4me2 (Soyer et al. 2015). GC content ranged from 31% to 58% with an average of 52% among windows (Table S2). TEs varied widely (0% to 100%) among windows with an average occupancy of 19%, while coding sequences occupied on average 43% of the windows (Table S2). The active histone mark H3K4me2, typically associated with promoters and enhancers of active genes, was the most frequently found mark across the genome, consistent with the high gene density. H3K4me2 was positively correlated with coding sequences (*r* = 0.71), while repressive histone marks (H3K9me3) were positively correlated with regions rich in TEs (*r* = 0.87) (Fig. 1b), especially DNA transposons (*r* = 0.28), LINEs (*r* = 0.51), and LTR retrotransposons (*r* = 0.65) (Fig. 1c, Fig. S2a). Since the presence of TEs was positively correlated with H3K27m3 (*r* = 0.49), we assessed whether the most recent TE activity (ie new insertions) was associated with variation in H3K27m3 among isolates. Using the newly generated ChIP-seq population survey for H3K27m3, we defined the H3K27m3 mean coverage and coefficient of variance (CoV) for each genomic window using data for all 45 field isolates. Regions of high H3K27m3 variation among isolates were positively correlated with newly inserted LTR retrotransposons (*r* = 0.36) (Fig. 1d). Topologically associated domains (TADs) described for the reference genome IPO323 were shown to be enriched in H3K27m3, in particular on accessory chromosomes (Glavincheska and Lorrain 2025). Based on H3K27m3 coverage across isolates, we classified TADs as consistently covered (>20% coverage in all isolates), uncovered (<20% in all isolates), or variable. Here, we observed that individual TADs located in both core and accessory chromosomes also exhibit variation in H3K27m3 coverage among isolates (Fig. S2b).

### Within-population Variation in H3K27m3 Gene Body Coverage

**Fig. 2.**
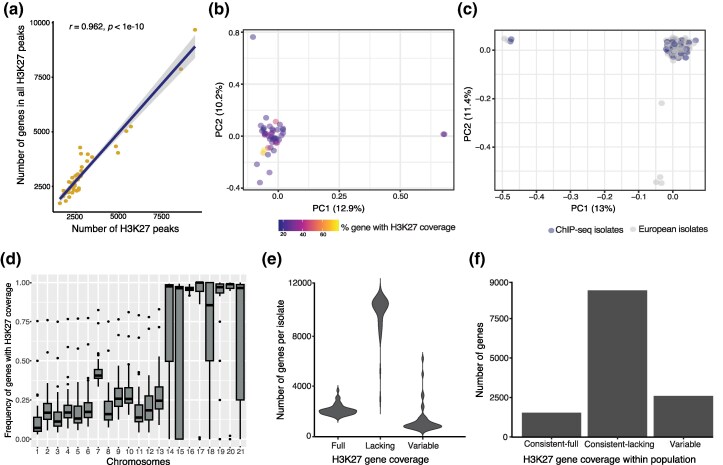
Genome-wide variation in H3K27m3 methylation marks covering gene bodies. a) Number of genome-wide genes overlapping with H3K27m3 peaks. Each data point represents an isolate. b) Principal component analysis of genetic variation in the population. Each data point represents an isolate. c) Principal component analysis of genetic variation in the study population, including representative European isolates. d) Density of H3K27m3 coverage across gene bodies on chromosomes within the population. e) Number of genes per isolate in each gene category defined by H3K27m3 coverage. f) The number of genes per category is defined by the variability or consistency of H3K27m3 coverage across the population.

We found substantial variation among isolates in the proportion of H3K27m3 marked genes (Fig. 2a), ranging from 14% to 75% of coverage. To assess whether this variation was associated with genetic variation, we performed a principal component analysis of genetic variants. Three isolates did not cluster with the remaining isolates. However, this separation was not explained by differences in H3K27me3 gene body coverage (Fig. 2b). Further analysis, including representative European isolates, showed that the genotypes fall well within the European genetic diversity (Fig. 2c). Marks were consistently higher for genes on accessory chromosomes compared to core chromosomes (Fig. 2d). Gene body coverage showed a strongly bimodal distribution of either no or complete H3K27m3 coverage (Fig. S3a). We classified H3K27m3 gene body coverage into 3 categories: no coverage (<20%), partial coverage (20% to 80%), and full coverage (>80%). A pattern was considered consistent if at least 80% of isolates showed the same coverage category; all other instances were classified as variable. We found that most of the genes lacked H3K27 coverage (75.7%) (Fig. 2e), with 69.2% of the genes revealing a consistent lack of H3K27m3 coverage across isolates (Fig. 2f). The remaining 11.4% and 19.4% of the genes showed consistent and variable gene body coverage by H3K27m3 marks within the population, respectively (Fig. 2f, Table S3). Genes highly expressed *in planta* were more consistently lacking H3K27m3 coverage (86.5%) compared to the other genes (71.6%) (Fig. S4a).

### Epigenomic Variation of Pathogenesis-related Genes and Gene Clusters in the Population

**Fig. 3.**
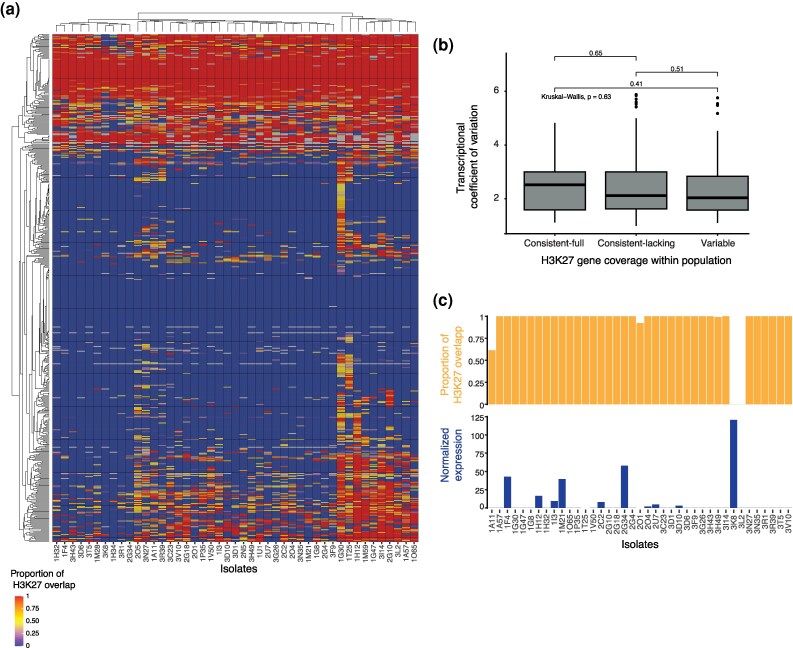
H3K27m3 gene body coverage variation among candidate effector genes. a) H3K27m3 profile in *Z. tritici* effector candidate genes. b) Transcriptional variation of effector genes across gene categories defined by H3K27m3 coverage within the population. c) *AvrStb6* H3K27m3 coverage and expression per isolate.

We analyzed H3K27m3 mark variation in gene bodies for 2 important categories of virulence-associated functions: candidate effector genes and gene clusters encoding secondary metabolite biosynthetic pathways. We found that most of the candidate effector genes (56.2%) showed a consistent lack in H3K27m3 coverage across the gene body, while 13.4% exhibited consistent complete coverage, and 30.4% showed variation in H3K27m3 gene body coverage among isolates (Fig. 3a). Considering only H3K27m3 peaks shared by both independent peak calling algorithms, 60.8% of candidate effector genes consistently lacked H3K27m3 gene body coverage, 28.1% exhibited variable coverage, and 11.1% showed consistent complete coverage (Table S3). Overall, these proportions indicate that the independent H3K27m3 peak calling approaches remain largely consistent even in regions encoding effectors. By analyzing the expression variation of candidate effector genes across all isolates, under the same nutrient-poor conditions used to obtain H3K27m3 profiles, we observed that expression variation was higher for effector genes consistently covered by H3K27 marks (Fig. 3b). However, differences in expression variation showed no correlation with differences in H3K27m3 effector gene coverage (*r* = −0.04) (Fig. 3b), suggesting that this facultative repressive marker might not act as a decisive factor for expression under the tested conditions. We investigated the epigenetic profile of the major effector gene encoding *AvrStb6*, a small, secreted protein triggering resistance responses in wheat cultivars carrying the *Stb6* gene in a gene-for-gene (GFG) interaction (Zhong et al. 2017). The large majority (82%) of the isolates showed complete coverage by H3K27m3 marks (Table S4). Although loss of *AvrStb6* was shown to occur at a low frequency in various populations (Sampaio et al. 2025), an incomplete H3K27m3 mark can also occasionally occur when the effector is present (Fig. S5a). Consistent coverage by H3K27m3 is tied to silencing of *AvrStb6*, resulting in absent or greatly reduced expression, but lack of H3K27m3 coverage was associated with both high and low *AvrStb6* expression (Fig. 3c).

We also investigated 5 candidate effector genes (Lapalu et al. 2025 Apr 28), which were previously shown to vary in their expression among isolates during infection (Palma-Guerrero et al. 2016). These include genes encoding a hydrophobin (ZtIPO323_12724), a DNase (_6487), a ribonuclease (_7456) cytotoxic against plants and microbes, a putative phytotoxin inducing necrosis and defense responses in plane trees (_8099) and a gene of unknown function (_4110). Most genes showed variation in H3K27m3 coverage among isolates (Fig. S5b), consistent with the observed expression variation. The only exception was ZtIPO323_6487 with consistent gene body coverage by H3K27m3 among isolates (Fig. S5b).

**Fig. 4.**
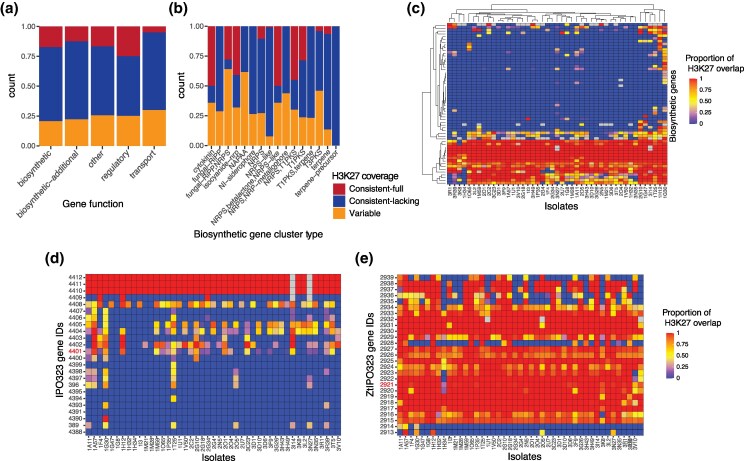
H3K27m3 gene body coverage of secondary metabolite gene clusters. a) H3K27m3 coverage profile by secondary metabolite gene functions. b) H3K27m3 coverage profile by biosynthetic gene cluster type. c) H3K27m3 profile of secondary metabolite core biosynthetic genes. d) H3K27m3 profile of polyketide synthase–encoding biosynthetic gene cluster. Gene ID 4401 is the core gene in the BGC. e) H3K27m3 profile of abscisic acid–encoding biosynthetic gene cluster. Gene ID 2921 is the core gene in the BGC.

To investigate variation in H3K27m3 gene body coverage of secondary metabolite gene clusters, we analyzed 788 genes forming 39 predicted gene clusters (Table S5). We found that 59.5% of all gene clusters consistently lack H3K27m3 marks among isolates, 24.9% of genes showed variable H3K27m3 coverage, and 15.6% were consistently covered by H3K27m3. Considering only peaks detected by both independent peak callers, 63.8% of all gene clusters consistently lacked H3K27m3 gene body coverage, 22.9% exhibited variable coverage, and 13.3% showed consistent complete coverage (Table S3). Genes encoding (core and additional) biosynthesis, regulatory, or transport functions exhibited similar levels of variation in H3K27m3 mark coverage, ranging between 20.7% and 30% among isolates (Fig. 4a). Most of the secondary biosynthetic gene clusters exhibited different H3K27m3 coverage patterns. In contrast, genes encoding terpene synthesis clusters (26 genes in 4 clusters) were consistently lacking H3K27m3 marks (Fig. 4b). Biosynthetic core genes also showed high variation in H3K27m3 coverage among isolates (Fig. 4c). For instance, a polyketide synthase (PKS) gene cluster shows variation in the H3K27m3 mark variation in both the biosynthetic and additional biosynthetic genes of the cluster (Fig. 4d). A PKS core gene (ZtIPO323_4401), which has been associated with antimicrobial activity against other fungi, was also showing a highly variable H3K27m3 profile (Zhang et al. 2018; Singh et al. 2022) (Fig. 4d). We observed no variation in H3K27m3 marks for a biosynthetic core gene linked to abscisic acid production (ZtIPO323_2921) experiencing strong upregulation during the transition from the biotrophic to the necrotrophic phase (Fig. 4e) (Palma-Guerrero et al. 2017).

## Discussion

We produced the first genome-wide assessment of population-scale variability in repressive histone modifications in a fungal plant pathogen. Our analyses show that gene presence and the activity of TEs are correlated with H3K27m3 marks across the genome. We also observed that the proportion of H3K27m3-marked genes varies among members of the same species and that genes on accessory chromosomes typically show higher proportions of marked genes. Approximately one-fifth of all genes show heritable variation in H3K27m3 marks even in a single population of the pathogen. Interestingly, we also found a large-scale repressive histone mark variation for genes important for pathogenicity (ie effector candidates and gene clusters involved in the biosynthesis of secondary metabolites).

Perturbations in the chromatin state of a chromosomal region can mediate transcriptional variation among individuals (Kasowski et al. 2013). TEs are often marked by repressive marks such as H3K9me3, acting as a powerful repressor of their transcription and transposition activity (Torres et al. 2020; Kramer et al. 2021). However, H3K27m3 is also positively correlated with increased TE transcription both in *Z. tritici* and the rice blast fungus *Magnaporthe oryzae* (W. Zhang et al. 2021a; Abraham et al. 2024). In *M. oryzae*, H3K27m3 was found broadly enriched across TE-rich regions, especially near effector genes (W. Zhang et al. 2021a). In *Z. tritici*, H3K27m3 marks are more abundant, particularly near DNA transposons, LINEs, and LTR retrotransposons. In *M. oryzae,* LINEs and LTR retrotransposons also showed H3K27me3 marks, but not for DNA elements (W. Zhang et al. 2021a). LTRs have been broadly found to be targeted by PRC2, responsible for catalyzing H3K27me3 (Hisanaga et al. 2023). Here, we show that regions with LTRs are indeed associated with H3K27m3 variation within the species, suggesting that the LTR targeting may be heterogeneous and possibly dynamic, given the ongoing activity by LTRs within the species (Baril et al. 2025).

Genes can also be marked by facultative repressive histone markers. Our within-species analyses showed that a substantial minority of all genes exhibited variation in H3K27me3 mark coverage among isolates, representing a substantial epigenomic variability. Considering the transcriptional silencing associated with H3K27m3 marks over the gene body (Pan et al. 2017), the observed H3K27m3 variability likely plays a role in gene expression variation observed within the species (Abraham and Croll 2023). Consistent with (Schotanus et al. 2015), we also observed a striking difference in H3K27m3 marks between core and accessory chromosomes. Genes located on accessory chromosomes were found to have a high proportion of H3K27m3 marks consistent with their likely dispensable roles. Furthermore, in *Z. tritici*, the loss of H3K27m3 increases the stability of some accessory chromosomes, suggesting an important role of H3K27m3 in genome integrity (Möller et al. 2019). Among core chromosomes, genes located on chromosome 7 showed comparatively higher H3K27m3 overlap with the gene body. This observation may be linked to the proposed origin of the right arm of chr 7 from an accessory chromosome (Schotanus et al. 2015). Moreover, chromosome 7 showed an enrichment of TEs compared to other core chromosomes. Flanking TEs may spread the repressive chromatin marks to the neighboring gene and downregulate gene expression (Soyer et al. 2014; Fouché et al. 2020). The low proportion of regulatory variants mapped for genes on chromosome 7 is consistent with the proposed role H3K27m3 plays on accessory chromosomes (Abraham and Croll 2023). Our study revealed intra-species variation in H3K27m3, suggesting that such epigenetic differences may contribute to the observed variation in gene expression across the population.

Histone modifications were widely studied in the context of pathogenicity-associated genes such as effector-encoding and secondary metabolite genes (Dubey and Jeon 2017; Dai et al. 2020). The epigenetic variation associated with pathogenicity-related genes within species could constitute adaptive genetic variation selectable for more optimal responses to environmental cues. We observed a high variability of H3K27m3 marks for candidate effector genes (30.4%) and secondary metabolite encoding genes (24.9%) in the population compared to background genes (19.4%). H3K27m3 also governs gene regulation as a response to various environmental stress factors. For example, in the rice blast fungus *M. oryzae,* effector genes marked by H3K27m3 during axenic growth were affected by chromatin dynamics and transcriptional variation during host infection (W. Zhang et al. 2021a). The associations of H3K27m3 with genes encoding proteinaceous and metabolic effectors or proteins involved in stress responses are also known from the fungal plant pathogen *Leptosphaeria maculans (Soyer et al. 2014).* The role of H3K27m3 in the regulation of secondary metabolite gene clusters in fungi has also been extensively studied (Collemare and Seidl 2019; Pfannenstiel and Keller 2019). Here, we observed that genes encoding biosynthesis (core and additional), regulatory, transport functions, and others exhibited similar levels of variation in H3K27 marks, ranging between 20.7% and 30%. This suggests that a local and histone-mediated regulation of such genes, apart from the transcription factors mediating global regulation of metabolite clusters (Collemare and Seidl 2019). We observed variable H3K27m3 profiles in the biosynthetic genes of the PKS secondary metabolite gene cluster (ZtIPO323_4401). The presence of a SNP located in the 3′UTR of this same gene has been previously associated with an antimicrobial active at least against the basidiomycete *Albatrellus confluence* (Zhang et al. 2018; Singh et al. 2022). Hence, histone methylation profiles and analyses of polymorphism associated with trait expression can be combined.

Further studies to understand the genetic basis of the H3K27m3 variation within species will provide a picture of the complex interplay of genetic and epigenetic variation in the PKS encoding secondary metabolite gene cluster. Our study established the first population-level histone methylation profile for a fungal species. We found extensive variation in H3K27m3 marks spanning a large part of the gene body. The epigenetic variation present within a single field population highlights the challenge to contain rapidly evolving pathogens and indicates an additional, non-genetic source of phenotypic plasticity that may influence disease dynamics and compromise the durability of disease management strategies.

## Materials and Methods

### Chromatin Immune-Precipitation and Sequencing

Isolates of Z. tritici (*n* = 45) were collected from an experimental wheat field planted with different cultivars in 2016 *(Karisto et al. 2018; Singh et al. 2021b)*. Isolates were grown for 10 d in a modified Vogel's (Minimal) Medium N with ammonium nitrate replaced by potassium nitrate and ammonium phosphate, without sucrose and agarose to induce hyphal growth *(Vogel 1956)*. We performed chromatin immunoprecipitation on the collected mycelia following (Soyer et al. 2015): 2.5 mL of 20% formaldehyde (final concentration ∼0.5%) was added directly to the flask and incubated for 15 min at room temperature while shaking (100 rpm). Formaldehyde was quenched by adding 2 mL of 2.5 M glycine, followed by centrifugation at 2,000 rpm for 5 min, and the pellet was washed with 1 X PBS. The resulting pellet (150 mg) was frozen in liquid nitrogen and homogenized using a mortar and pestle. Ice-cold lysis or chromatin buffer was added in a ratio of ∼5 µL chromatin buffer to 1 mg of pellet. Micrococcal nuclease (MNase, #M02479; NEB) was added to the reaction and incubated for 10 to 20 min in a 37 °C water bath and mixed every 2 min by inversion. To stop the reaction, 4 µL of 0.5 M Na-EGTA (pH 8.0) was added and the samples were placed on ice. Samples were mixed and centrifuged at 4,000 rpm for 5 min at 4 °C. Then, 800 µL supernatant was transferred to a fresh tube, and nonspecific proteins were pre-cleared with magnetic Dynabeads (Invitrogen) by incubation at 4 °C on a rotator for 1 h. Then, samples were centrifuged at 5,000 rpm for 1 min. The histone H3K27me3 antibody (pAb) from Active Motif (Cat. No. 39055) was added (5 µL) to each sample tube and incubated overnight at 4 °C on a rotator. After the overnight incubation, 20 µL of magnetic Dynabeads were added and incubated for 2 h at 4 °C on a rotator to allow for antibody binding. Samples were placed on a magnetic rack, and the supernatant was discarded. The pellet was washed with 1 mL of cold ChIP lysis buffer, 1 mL of ice-cold LiCl, and 1 mL of ice-cold TE buffer. Next, 63 µL of 65 °C TE buffer with sodium dodecyl sulfate was added and incubated for 10 min at 65 °C, resulting in the elution of DNA from the beads. The elution was repeated with another 63 µL of warm TES and supernatants were pooled. Chromatin crosslinks were reversed by incubating samples for 6 to 16 h in a 65 °C incubator. To the de-crosslinked samples, 125 µL of water and 1.9 µL of 20 mg/mL RNase A were added, and tubes were incubated at 50 °C for 2 h. Subsequently, 9.5 µL of 20 mg/mL proteinase K was added and tubes were incubated for another 2 h at 50 °C. The resulting supernatant was cleaned and eluted in 30 µL of nuclease-free water using Wizard SV Gel and PCR Clean-Up System. The ChiP-sequencing library was prepared for sequencing and analyzed using a NovaSeq 6000 in paired-end mode with a read length of 150 bp.

### Quality Control and Peak Calling

Raw ChIP-seq sequencing data were checked for quality using FastQC v0.12.1 (Andrews 2010) and trimmed with Trimmomatic v0.39 (Bolger et al. 2014) to remove adapter sequences and low-quality reads based on the following parameters: ILLUMINACLIP: TruSeq3-PE.fa:2:30:10 LEADING:3 TRAILING:3 SLIDING WINDOW:4:15 MINLEN:36. Trimmed sequences were aligned to the *Z. tritici* IPO323 reference genome (Lapalu et al. 2025 Apr 28) using Bowtie2 v2.4.1 (Langmead and Salzberg 2012) with the option --very-sensitive-local. Duplicated sequences were tagged and removed using the Picard MarkDuplicates function v2.27.4 (http://broadinstitute.github.io/picard). ChIP-seq data quality was analyzed using the SSP (strand-shift profile) tool v1.1.0 (https://github.com/rnakato/SSP) with short background length option to quantify the signal-to-noise ratio (NSC), response signal correlation (RSC), and the mapped-read distribution throughout the genome (Bu). We used the Picard tool EstimateLibraryComplexity to calculate the library complexity (ie the non-redundant read fraction per 10 million mapped reads). The FRiP score (fraction of reads in peaks) was calculated using SAMtools v1.6 (Danecek et al. 2021) to count total mapped reads and BEDTools v2.30.0 (Quinlan and Hall 2010) to intersect and quantify reads overlapping peak regions. Peak calling was performed using the findPeaks function in the software package Homer (v2.6.6) (http://homer.ucsd.edu/homer/ngs/peaks.html) with a peak calling size of 800 base pairs, which specifies the width of peaks that will form the basic building blocks of peaks in the regions (Heinz et al. 2010). The minimum distance between adjacent peaks was set to 800 bp. ChIP-seq peaks were annotated for nearby gene features using BEDTools (v2.30.0) (Quinlan and Hall 2010), and gene models predicted for the IPO323 genome by Lapalu et al. (Lapalu et al. 2025 Apr 28) were used. MACS3 (v3.0.3) (Feng et al. 2011) was used with the –broad option as an independent peak caller employing a distinct background modeling strategy to assess consistency of HOMER peak-calling results in the absence of an input control. Overlap between HOMER and MACS3 peaks was quantified as the percentage of overlapping base pairs using BEDTools. (v2.30.0) (Quinlan and Hall 2010).

### Genomic Window Analyses

We analyzed genomic factors associated with previously generated H3K27me3, H3K4me2, and H3K9me3 ChIP-seq datasets aligned to the IPO323 reference genome (Soyer et al. 2015). We defined windows as non-overlapping 10 kb intervals using BEDTools v2.30.0 (Quinlan and Hall 2010) and we calculated the percentage of base pairs covered by ChIP-seq peaks within each 10 kb bin. For correlation analyses, the percentage of base pairs covering each bin was also calculated for other available parameters from the reference genome (IPO323): ie annotated genes (Lapalu et al. 2025 Apr 28); TEs detected using a curated *Z. tritici* TE consensus library (Baril and Croll 2023); and recently inserted TEs (Baril et al. 2025); GC content per 10 kb bin calculated using geecee v6.6.0.0 (https://www.bioinformatics.nl/cgi-bin/emboss/geecee). We also analyzed 3D genome organization features such as TADs (Glavincheska and Lorrain 2025 May 17) to assess H3K27m3 mark distribution. We considered H3K27m3 TAD coverage <20% in all isolates as no coverage and >20% as consistent coverage. Variable-switch was defined as isolates exhibiting TADs with extreme coverage variation (below 20% and above 80% coverage among isolates, respectively), whereas variable-nonswitch referred to isolates having TADs spanning the full H3K27m3 coverage spectrum, including at least one isolate with no coverage.

We further explored genomic features associated with H3K27m3 variation by ChIP-seq read coverage generated from the 45 field isolates. We used the same non-overlapping 10 kb intervals for summary statistics. To discard possible false negative ChIP-seq results caused by segmental deletions, we used previously generated copy number variation (CNV) data (Tralamazza et al. 2024) obtained in intervals of 1 kb using GATK CNV caller v4.1.9.0 (Babadi et al. 2018). We considered genomic segment to be absent in a particular isolate if half or more of it was called as missing by the CNV calling step. For each isolate, we calculated the percentage of base pairs covered by H3K27m3 peaks per 10 kb bin. We then computed the mean and coefficient of variation (CoV) across isolates to quantify average enrichment and variability. Then we used isolate-specific new TE insertion data and summarized evidence for non-reference insertions per 10 kb bin (Baril and Croll 2023). The number of non-reference TE insertions across all isolates per bin was summed and used as a parameter in correlation analyses.

### Population Genetic Diversity

Genetic diversity among isolates was assessed by a principal component analysis (PCA) using genome-wide SNPs called both for the same isolates as used for ChIP-seq and additional, representative European isolates *(Feurtey et al. 2023)*. SNPs were filtered for minor allele frequency (MAF) ≥ 0.05 and only biallelic SNPs were kept for further analysis. To control for missing data, only SNPs called in at least 90% of isolates were kept for further analysis using vcftools *(Danecek et al. 2011)*. In addition, variants were thinned to one SNP for every 1 kb to reduce overrepresentation of highly polymorphic genomic regions. The filtered VCF was converted to PLINK binary format using PLINK v1.9 *(Purcell et al. 2007)*. PCA was computed using the –pca function in PLINK and visualized using ggplot2 (v3.5.2).

### Population Variation Analyses

Individual gene loci were analyzed for evidence of gene deletions to remove erroneous calls of H3K27m3 peak variation caused by the lack of the underlying sequence in some isolates. A gene was considered deleted in a particular isolate if half or more contain null CNV calling, as described above. RNA sequencing data were accessed for all 45 *Z. tritici* isolates (Abraham and Croll 2023). The RNA-seq data were generated from isolates grown in modified Vogel's Medium N (Minimal), replacing ammonium nitrate with potassium nitrate and ammonium phosphate. The media was devoid of any sucrose and agarose to induce hyphal growth. The NucleoSpin RNA Plant and Fungi kit were used to extract total RNA from filtered mycelium after 10 to 15 d. An Illumina HiSeq 4000 was used to sequence TruSeq stranded mRNA libraries with 150 bp inserts in single-end mode. We checked RNA sequences for quality using FastQC v0.11.5 (Andrews 2010) and trimmed using Trimmomatic v0.36 (Bolger et al. 2014) to remove adapter sequences and low-quality reads. We aligned trimmed sequences to the *Z. tritici* reference genome using HISAT2 v2.1.0 (Y. Zhang et al. 2021b) with the parameter “--RNA-strandedness reverse.” The reads mapped to gene models were counted using the QTLtools v1.1 (Delaneau et al. 2017) in --Quan mode. Normalization of the counts was done using the --rpkm option implemented in QTLtools. Genes upregulated during host infection were selected according to previous host infection transcriptome analyses obtained from *Z. tritici* isolates with high-quality genomes (Palma-Guerrero et al. 2016).

To address H3K27m3 gene coverage within the population, we considered H3K27m3 gene body coverage below 20% as no evidence for coverage, above 80% as full coverage, and intermediate percentages of overlap of H3K27m3 marks as partial coverage. Consistent full and lacking coverage was considered if at least 80% of the isolates exhibited the given pattern. The remaining were considered variable.

### Gene Function Enrichments

Effectors were predicted on the secretome using EffectorP v2.0 (Sperschneider and Dodds 2022) based on *Z. tritici* IPO323 reference genome (Lapalu et al. 2025 Apr 28). Secondary metabolites gene clusters were predicted using antiSMASH v.5.0 (Blin et al. 2019) also based on the reference genome.

## Supplementary Material

evag118_Supplementary_Data

## Data Availability

RNA-seq and ChIP-seq datasets are available from the NCBI Sequence Read Archive accession numbers PRJNA650267 and PRJNA1321741, respectively.
