# Supplementary material for: Population-level Variability in Genome-wide Repressive Histone Marks in a Fungal Wheat Pathogen
Source: Genome Biol Evol. 2026 May 8;18(5):evag118. doi: 10.1093/gbe/evag118 (PMC13221638; doi:10.1093/gbe/evag118)
Supplement: evag118_Supplementary_Data [file evag118_supplementary_data.zip › Supplementary_Figures.pdf]

Supplementary Figures

Abraham et al.

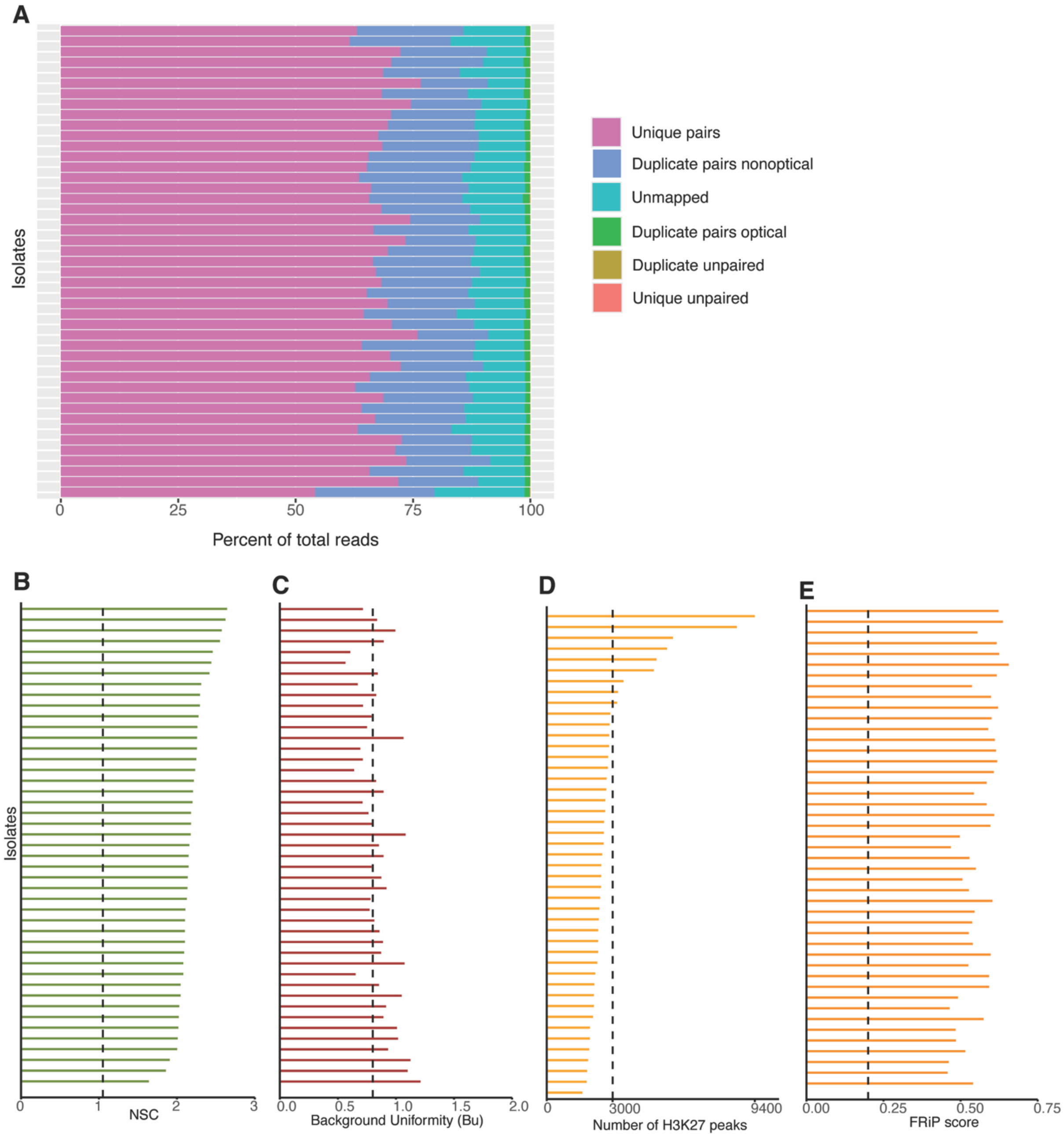

Supplementary Figure S1: A) Percentage of total reads mapped to the *Z. tritici* reference genome IPO323. B) Normalized strand cross-correlation coefficient (NSC) of aligned ChIP-seq reads. C) Background uniformity (Bu) of aligned ChIP-seq reads. D) Number of H3K27 peaks among isolates in the population. E) Fraction of reads in H3K27 peaks (FRiP score).

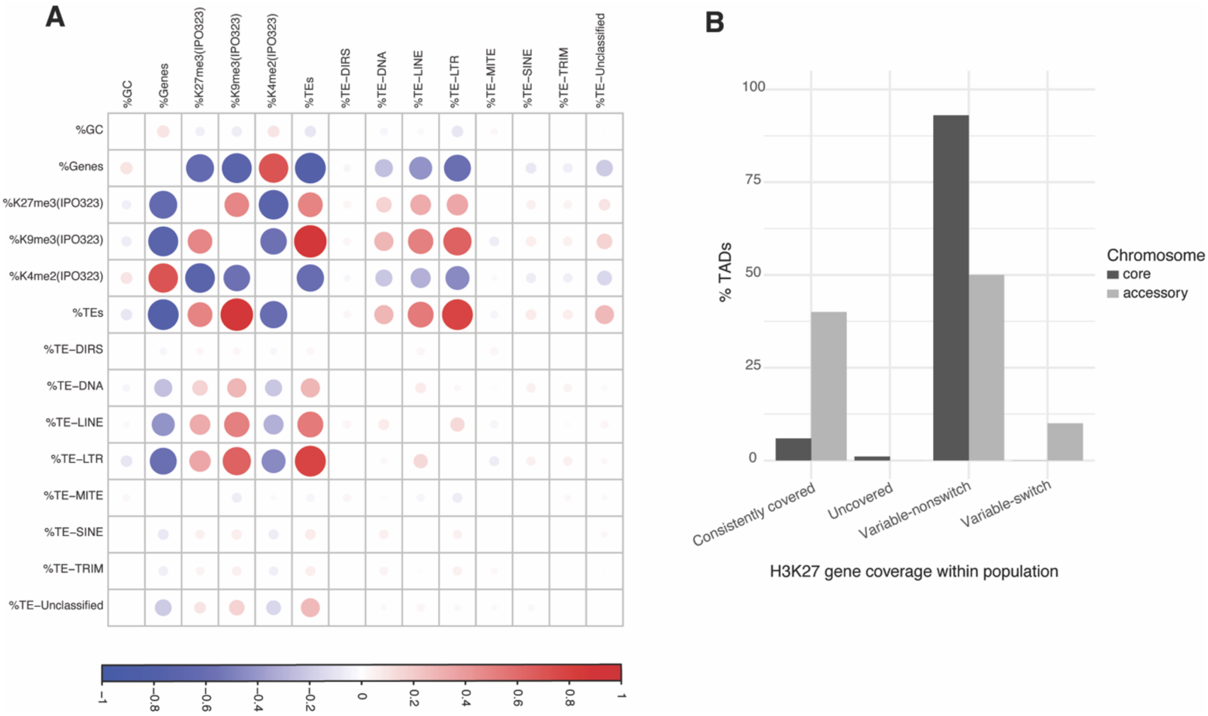

Supplementary Figure S2: A) Correlation plot between the percentage of 10-kb bins covered by GC, H3K27, H3K9, H3K4, genes, and TEs in IPO323 *Z. tritici* reference genome. B) Number of Topologically Associating Domains (TADs) in each H3K27 TAD coverage within population.

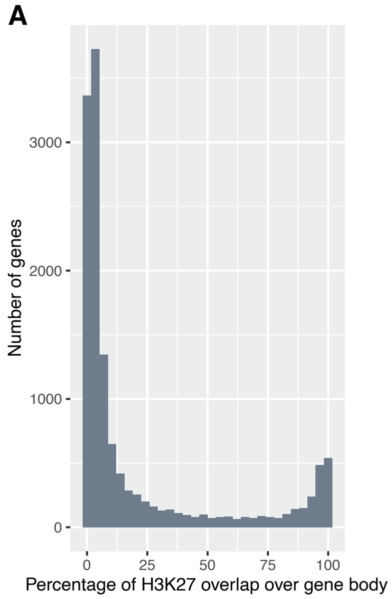

Supplementary Figure S3: A) Distribution of H3K27 mark coverage across genes.

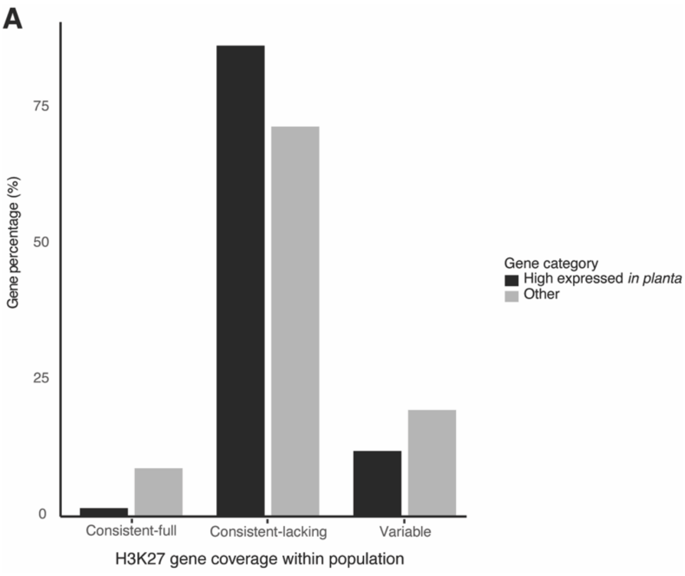

Supplementary Figure S4: A) Percentage of upregulated genes during host infection in each gene category defined by H3K27m3 coverage.

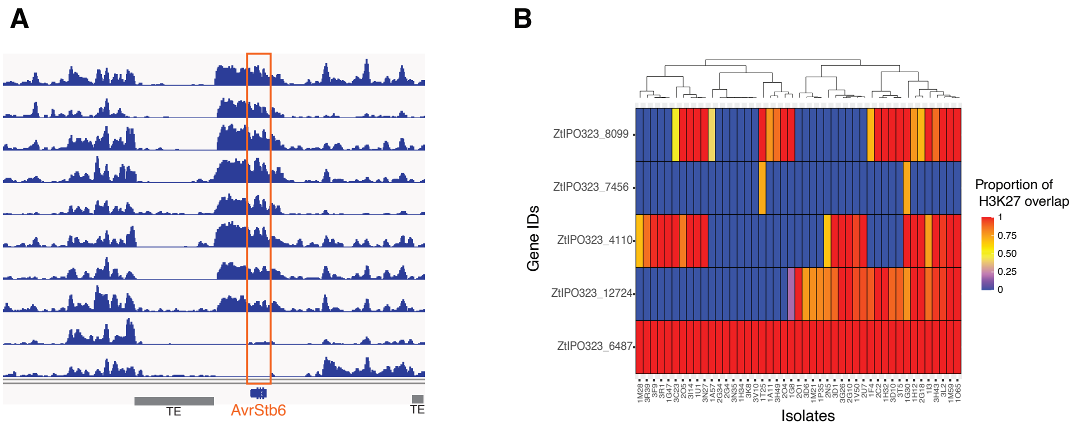

Supplementary Figure S5: A) H3K27m3 mark profile in *AvrStb6* gene among a subset of isolates in the population. B) H3K27 m3 coverage over candidate effector genes with variable expression over infection.
